# Differential Expression of Super-Enhancer-Associated Long Non-coding RNAs in Uterine Leiomyomas

**DOI:** 10.1007/s43032-022-00981-4

**Published:** 2022-05-31

**Authors:** Tsai-Der Chuang, Derek Quintanilla, Drake Boos, Omid Khorram

**Affiliations:** Department of Obstetrics and Gynecology, Harbor-UCLA Medical Center and The Lundquist Institute, Torrance, CA 90502 USA

**Keywords:** SE-lncRNA, Leiomyoma, Fibroid, MED12 mutation, Race

## Abstract

Super-enhancer-associated long non-coding RNAs (SE-lncRNAs) are a specific set of lncRNAs transcribed from super-enhancer (SE) genomic regions. Recent studies have revealed that SE-lncRNAs play essential roles in tumorigenesis through the regulation of oncogenes. The objective of this study was to elucidate the expression profile of SE-lncRNAs with concurrent assessment of associated mRNAs in leiomyomas and paired myometrium. Arraystar SE-lncRNAs arrays were used to systematically profile the differentially expressed SE-lncRNAs along with the corresponding SE-regulated protein coding genes in eight leiomyomas and paired myometrium. The analysis indicated 7680 SE-lncRNAs were expressed, of which 721 SE-lncRNAs were overexpressed, while 247 SE-lncRNAs were underexpressed by 1.5-fold or greater in leiomyoma. Thirteen novel SE-lncRNAs and their corresponding protein coding genes were selected, and their expression was confirmed in eighty-one paired leiomyoma tissues by quantitative real-time PCR. The thirteen pairs of SE-lncRNAs and their corresponding protein coding genes included RP11-353N14.2/CBX4, SOCS2-AS1/SOCS2, RP1-170O19.14/HOXA11, CASC15/PRL, EGFLAM-AS1/EGFLAM, RP11-225H22/NEURL1, RP5-1086K13.1/CD58, AC092839.3/SPTBN1, RP11-69I8.3/CTGF, TM4SF1-AS1/TM4SF1, RP11-373D23/FOSL2, RP11-399K21.11/COMTD1, and CTB-113P19.1/SPARC. Among these SE-lncRNAs, the expression of SOCS2-AS1/SOCS2, RP11-353N14.2/CBX4, RP1-170O19.14/HOXA11, and RP11-225H22/NEURL1 was significantly higher in African Americans as compared with Caucasians. The expression of RP11-353N14.2/CBX4, SOCS2-AS1/SOCS2, CASC15/PRL, and CTB-113P19.1/SPARC was significantly higher in tumors with MED12-mutation-positive as compared with MED12-mutation-negative tumors. Collectively, our results indicate that the differential expression of SE in leiomyomas is another mechanism contributing to dysregulation of protein coding genes in leiomyomas and that race and MED12 mutation can influence the expression of a select group of SE.

**Supplementary Information:**

The online version contains supplementary material available at 10.1007/s43032-022-00981-4.

## Introduction

Uterine leiomyomas, also known as uterine fibroids, are common benign uterine smooth muscle tumors with an unknown etiology affecting up to 40–70% of reproductive-aged women. Some women with uterine leiomyomas are asymptomatic, while others may have abdominal and pelvic pain, excessive menstrual bleeding, and infertility depending on the location of the leiomyomas [1]. Leiomyomas are considered a fibrotic disorder and are characterized by excess accumulation of extracellular matrix (ECM), increased cell proliferation, and inflammation [2–4]. Ovarian steroids stimulate the growth and progression of these tumors [1]. High-throughput sequencing and proteomic studies have demonstrated altered expression of protein-coding genes which are critical in leiomyoma pathogenesis. Furthermore, these tumors are characterized by genetic heterogeneity and associated with chromosomal re-arrangements and mutations [5–7]. The driver mutations critical to leiomyoma development and progression include MED12 (mediator complex subunit 12), HMGA2 (high mobility group AT-hook 2), FH (fumarate hydratase), and COL4A5/6 (collagen type IV, alpha 5, and alpha 6) [8–11]. Recent studies have focused on the role of MED12 mutations in the pathophysiology of these tumors [12–15]. Somatic MED12 mutation in exon 2 occurs at a frequency of up to 80% and has a functional role in leiomyoma initiation and progression potentially through abnormal activation of Wnt/β-catenin signaling, sex steroid receptor signaling, fibrosis-associated gene expression, and cell proliferation [12–15].

In addition to aberrant expression of protein-coding genes and genetic heterogeneity, our group and others have reported that leiomyomas as compared with matched myometrium have differential expression of microRNAs (miRNAs), such as let7, miR-21, miR-29, miR-200c, and miR-93 [16–19]. These miRNAs target the transcription and translation of genes which are functionally associated with cellular growth and transformation, inflammatory responses, and ECM accumulation [16–19]. Our group has focused on the role of long non-coding RNAs (lncRNAs) in leiomyomas pathogenesis [20–22]. LncRNAs are single-strand RNA molecules with > 200 nucleotides. They are classified in five different groups based on their transcription from different genomic loci (sense, antisense, bidirectional, intergenic and intronic) [23–28]. LncRNAs are expressed in a cell- and tissue-specific manner and, through their interaction with miRNAs and mRNAs, induce epigenetic modifications or act as “molecular sponge” for miRNAs [23–26, 29]. Recent evidence using microarray and next-generation RNA sequencing has shown dysregulation in the expression of lncRNAs in leiomyomas [20, 30]. A special class of long non-coding RNAs which are transcribed from super-enhancer (SE) genomic loci are super-enhancer lncRNAs (SE-lncRNAs), which form RNA:DNA:DNA triplex with multiple anchor DNA sites within the super-enhancers [31–33]. SE-lncRNAs are a novel class of RNA regulators which can activate the expression of nearby genes through various mechanisms including recruitment of transcription factors to the super-enhancer loci, chromatin structure modification, chromatin looping, loading RNA polymerase II, and release of transcriptional repressor [31–33]. SE-lncRNAs can transfer from their site of expression and interact with different locations across multiple chromosomes to trans-activate target genes [31, 34].

The objective of this study was to determine the expression profile of SE-lncRNAs in leiomyomas as compared to matched myometrium which has not been done to date. We hypothesized that differential expression of SE-lncRNAs in leiomyomas could influence the expression of nearby protein coding genes which are pivotal to leiomyoma pathogenesis. To address this objective, we analyzed the expression profile of SE-lncRNAs in eight leiomyomas and paired myometrium using Arraystar Human SE-lncRNA Array (Arraystar, Rockville, MD, USA). We selected and further analyzed thirteen SE-lncRNAs and their corresponding protein coding genes which were differentially expressed in leiomyoma using quantitative real-time PCR (qRT-PCR) in different races and in leiomyomas with and without MED12 mutation. The selected SE-lncRNAs included RP11-353N14.2/CBX4 (chromobox 4), SOCS2-AS1 (SOCS2 antisense RNA 1)/SOCS2 (suppressor of cytokine signaling 2), RP1-170O19.14/HOXA11 (homeobox A11), CASC15 (cancer susceptibility 15)/PRL (prolactin), EGFLAM-AS1 (EGFLAM antisense RNA 1)/EGFLAM (EGF like, fibronectin type III and laminin G domains), RP11-225H22/NEURL1 (neuralized E3 ubiquitin protein ligase 1), RP5-1086K13.1/CD58 (CD58 molecule), AC092839.3/SPTBN1 (spectrin beta, non-erythrocytic 1), RP11-69I8.3/CTGF (connective tissue growth factor), TM4SF1-AS1 (TM4SF1 antisense RNA 1)/TM4SF1 (transmembrane 4 L six family member 1), RP11-373D23/FOSL2 (FOS like 2, AP-1 transcription factor subunit), RP11-399K21.11/COMTD1 (catechol-O-methyltransferase domain containing 1), and CTB-113P19.1/SPARC (secreted protein acidic and cysteine-rich).

## Materials and Methods

### Myometrium and Leiomyoma Tissue Collection

Portions of uterine leiomyomas and paired myometrium were obtained from patients (*n* = 81) who were not taking any hormonal medications for at least 3 months prior to surgery at Harbor-UCLA Medical Center. Prior approval from the Institutional Review Board (#036,247) at the Lundquist Institute at Harbor-UCLA Medical Center was obtained. Informed consent was obtained from all the patients participating in the study prior to surgery. The leiomyomas used in this study ranged in size from 3 to 5 cm in diameter and were intramural. The paired tissues were obtained from Caucasians (*n* = 12), African Americans (*n* = 25), Hispanics (*n* = 37), and Asians (*n* = 7). The mean age of patients was 45 ± 5.3 years with a range of 30–54 years. The MED12 mutation status was determined by PCR amplification and Sanger sequencing. Of the specimens sequenced, 52 leiomyomas had the MED12 mutations (52/81 pairs; 64.2%) with no mutations in the myometrium. The eight pairs of tissues used for SE-lncRNA microarray studies were from four Caucasians and four African Americans with two tumors being MED12-mutation-positive and two MED12-mutation-negative in each racial group. The tissues were snap frozen and stored in liquid nitrogen for further analysis as previously described [35, 36].

### MED12 Mutation Analysis

Genomic DNA from leiomyomas and paired myometrial specimens was extracted from 100 mg of freshly frozen tissue using MagaZorb DNA Mini-Prep Kit (Promega, Madison, WI) according to the manufacturer’s protocol. PCR amplification and Sanger sequencing (Laragen Inc. Culver City, CA) was performed to investigate the MED12 exon 2 mutations using the primer sequences in the 5’–3’ direction: sense, GCCCTTTCACCTTGTTCCTT, and antisense, TGTCCCTATAAGTCTTCCCAACC. PCR products were sequenced using Big Dye Terminator v.3.1 sequencing chemistry, and the sequences were analyzed with the Software ChromasPro 2.1.8 and compared with the MED12 reference sequence (NG_012808 and NM_005120).

### SE-lncRNA Microarray Analysis

The microarray and bioinformatic analyses were conducted by Arraystar Company. Briefly, each sample was amplified and transcribed into fluorescent cRNA along the entire length of the transcripts without 3’ bias utilizing a random priming method (Arraystar Flash RNA Labeling Kit, Arraystar). The labeled cRNAs were hybridized onto the Human Super-Enhancer LncRNA Microarray (8 × 15 K, Arraystar). After washing the slides, the arrays were scanned by the Agilent Scanner G2505C. Quantile normalization and subsequent data processing were performed using GeneSpring GX v12.1 software package (Agilent Technologies) and adjusting batch effects in expression data using empirical Bayes methods. Hierarchical clustering was performed to show the distinguishable expression of SE-lncRNAs and associated mRNAs among those samples. The associated mRNAs analyzed either overlapped or were within 50 kb of the SE-lncRNA transcription start site. The altered expression of SE-lncRNAs and associated mRNAs with statistical significance was identified through Volcano filtering between leiomyomas group and matched myometrium group. Gene ontology (GO) and Kyoto Encyclopedia of Genes and Genomes (KEGG) analysis were used to determine the roles of the differentially expressed associated mRNAs in biological pathways. Pathway-gene network (Path-Net) was constructed to illustrate the relationship between significantly affected pathways and differentially expressed SE-associated mRNAs. The SE-lncRNA microarray data was deposited in Gene Expression Omnibus (GEO) database with accession number (GSE193320).

### RNA Isolation and qRT-PCR Analysis

Total RNA was extracted from leiomyoma and matched myometrium using TRIzol (Thermo Fisher Scientific Inc., Waltham, MA). RNA concentration and integrity was determined using a Nanodrop 2000c spectrophotometer (Thermo Scientific, Wilmington, DE) and Agilent 2100 Bioanalyzer (Agilent Technologies, Santa Clara, CA) according to manufacturer’s protocols [37, 38]. Two μg of RNA was reverse transcribed using random primers for selected lncRNAs and their neighboring genes according to the manufacturer’s guidelines (Applied Biosystems, Carlsbad, CA). Quantitative RT-PCR was carried out using SYBR gene expression master mix (Applied Biosystems). Reactions were incubated for 10 min at 95 °C followed by 40 cycles for 15 s at 95 °C and 1 min at 60 °C. The levels of lncRNAs and their neighboring genes were quantified using Invitrogen StepOne System with FBXW2 (F-box and WD repeat domain containing 2) used for normalization [39]. All reactions were run in triplicate, and relative mRNA expression was determined using the comparative cycle threshold method (2-ΔΔCq), as recommended by the supplier (Applied Biosystems). The primer sequences in the 5’–3’ direction used are listed in supplementary Table 1.

### Statistical Analysis

Throughout the text, results are presented as mean ± SEM and analyzed by PRISM software (GraphPad, San Diego, CA). Dataset normality was determined by the Kolmogorov–Smirnoff test, Shapiro–Wilk test, D’Agostino and Pearson test, and Anderson–Darling test. The data presented in this study was not normally distributed, and therefore, non-parametric tests were used for data analysis. Comparisons involving two groups were analyzed using the Wilcoxon matched pairs signed rank test (Fig. 5) or Mann–Whitney test (Figs. 6 and 7) as appropriate. Statistical significance was established at *P* < 0.05.

## Results

### Expression of SE-lncRNAs and Their Corresponding Protein Coding Genes in Leiomyoma and Matched Myometrium

**Fig. 1:**
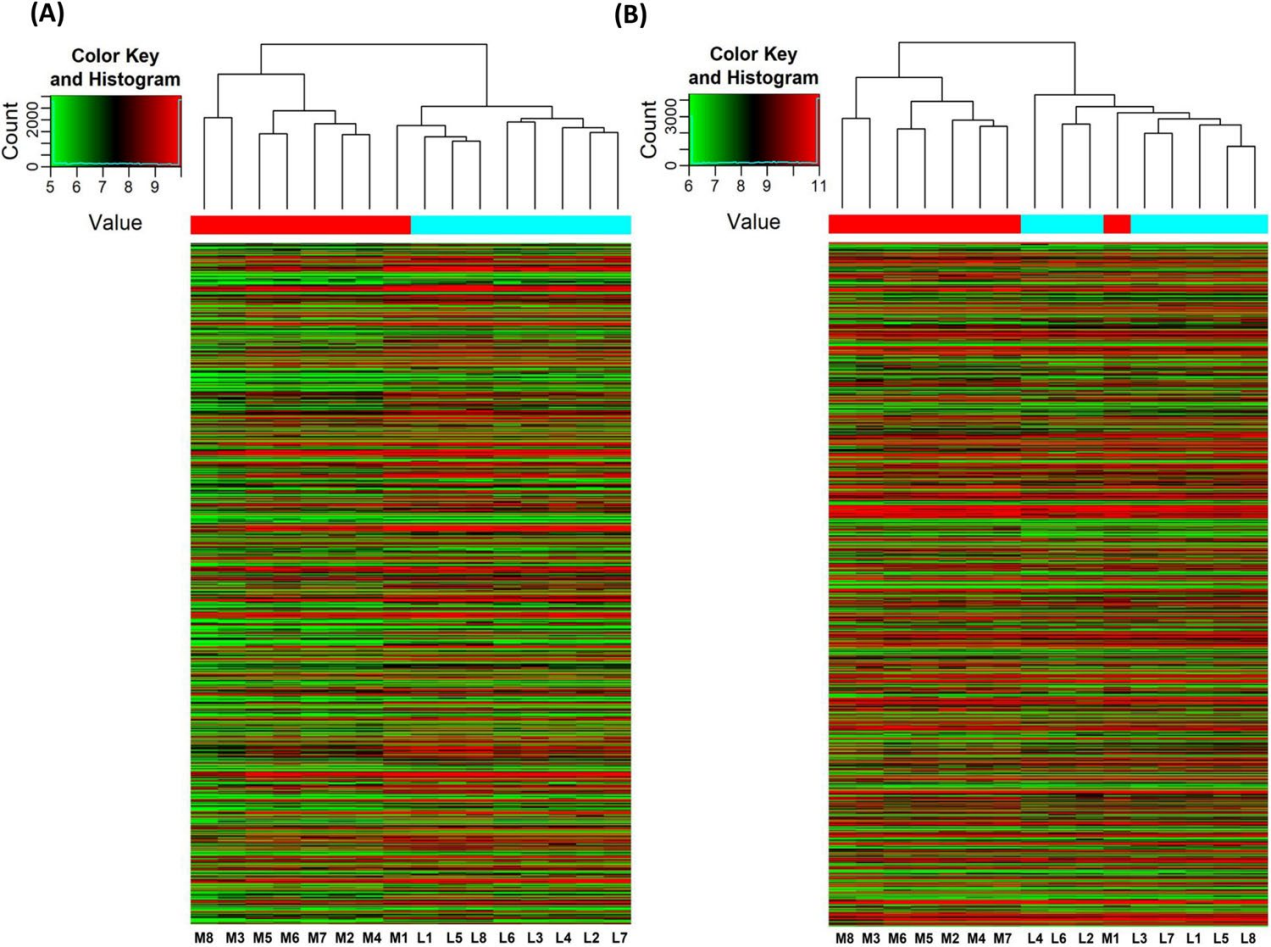
Hierarchical clustered heatmap analysis of all the differentially expressed SE-lncRNAs (**A**) and SE-associated mRNAs (**B**) in eight paired leiomyoma and matched myometrium. The clustering analysis was performed by Cluster 3.0 software with average linkage clustering algorithm and visualized by TreeView. “Red” and “green” indicate high relative expression and low relative expression (fold change ≥ 1.5, *P* < 0.05)

**Fig. 2:**
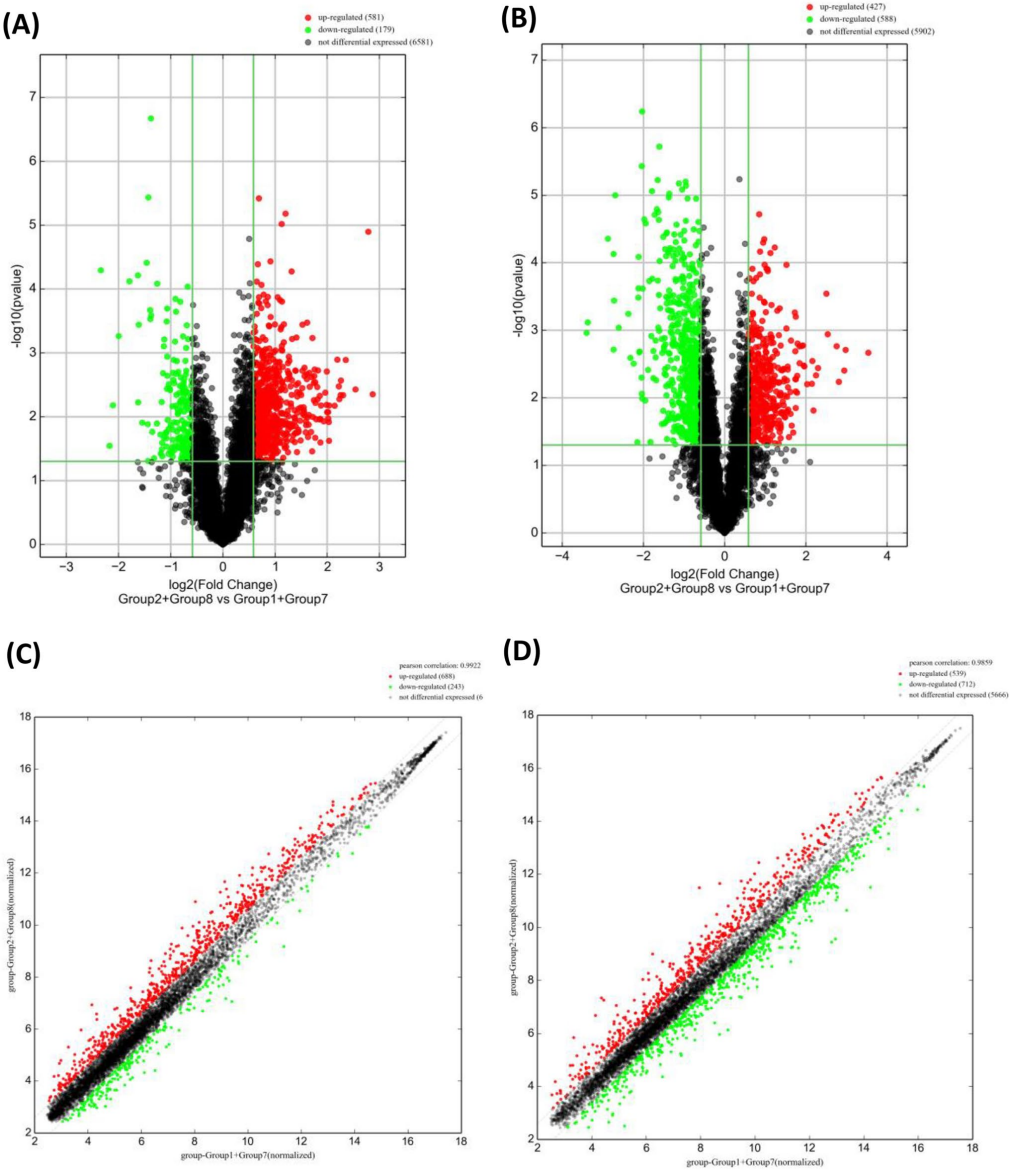
Volcano plots (**A**–**B**) and scatter plots (**C**–**D**) of aberrantly expressed SE-lncRNA (**A** and **C**) and SE-associated mRNA (**B** and **D**) in eight paired leiomyoma and matched myometrium. The red and green points correspond to 1.5-fold upregulation and downregulation (fold change ≥ 1.5, *P* < 0.05)

**Fig. 3:**
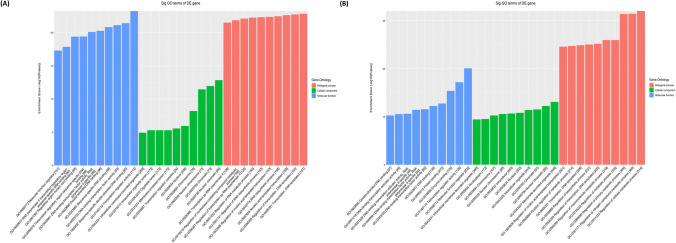

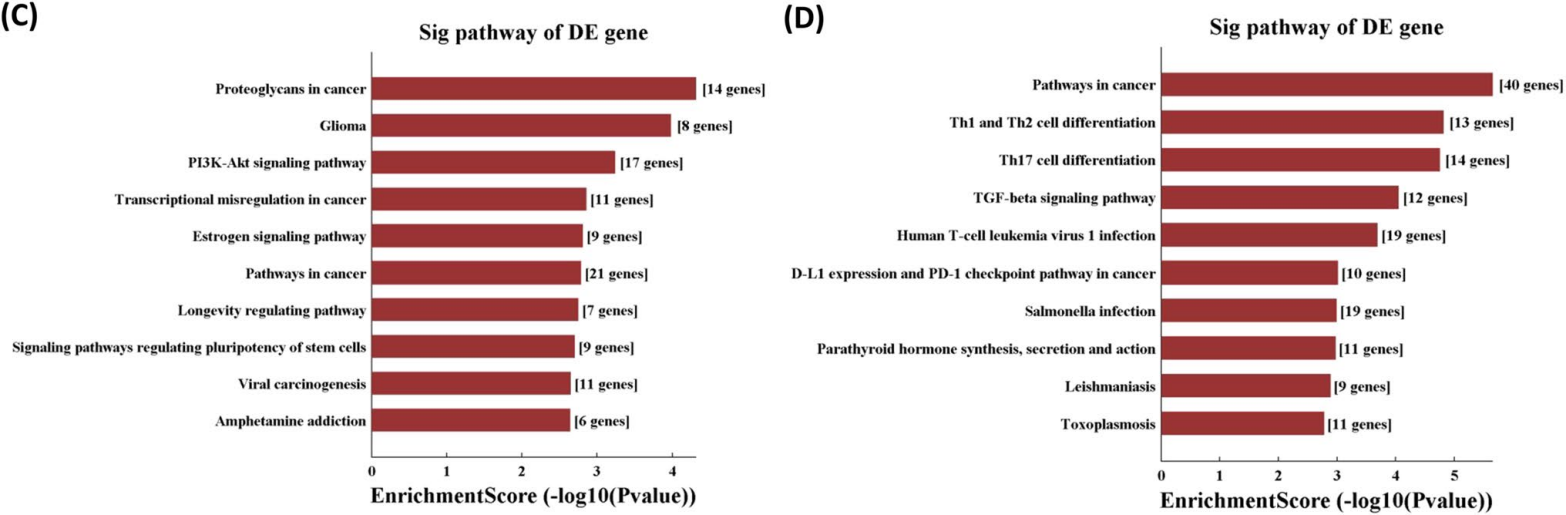
GO analysis (**A**–**B**) and KEGG pathway analysis (**C**–**D**) of differentially expressed (DE) genes in eight paired leiomyoma and matched myometrium. (**A**–**B**) GO analysis of up (**A**)- and down (**B**)-regulated SE-associated mRNAs with the top ten significantly enrichment score in the categories of biological processes, cellular components, and molecular functions as represented on the *x*-axis. The 10 items with the smallest values in each category were selected for graphic display. (**C**–**D**) KEGG pathway enrichment analysis of up (**C**)- and down (**D**)-regulated SE-associated mRNAs with the top ten significantly enrichment score. –Log10(*P* value), negative logarithm of the *P* value, is represented on the *x* axis. Larger –Log10(*P* value) displays a smaller *P* value

**Fig. 4:**
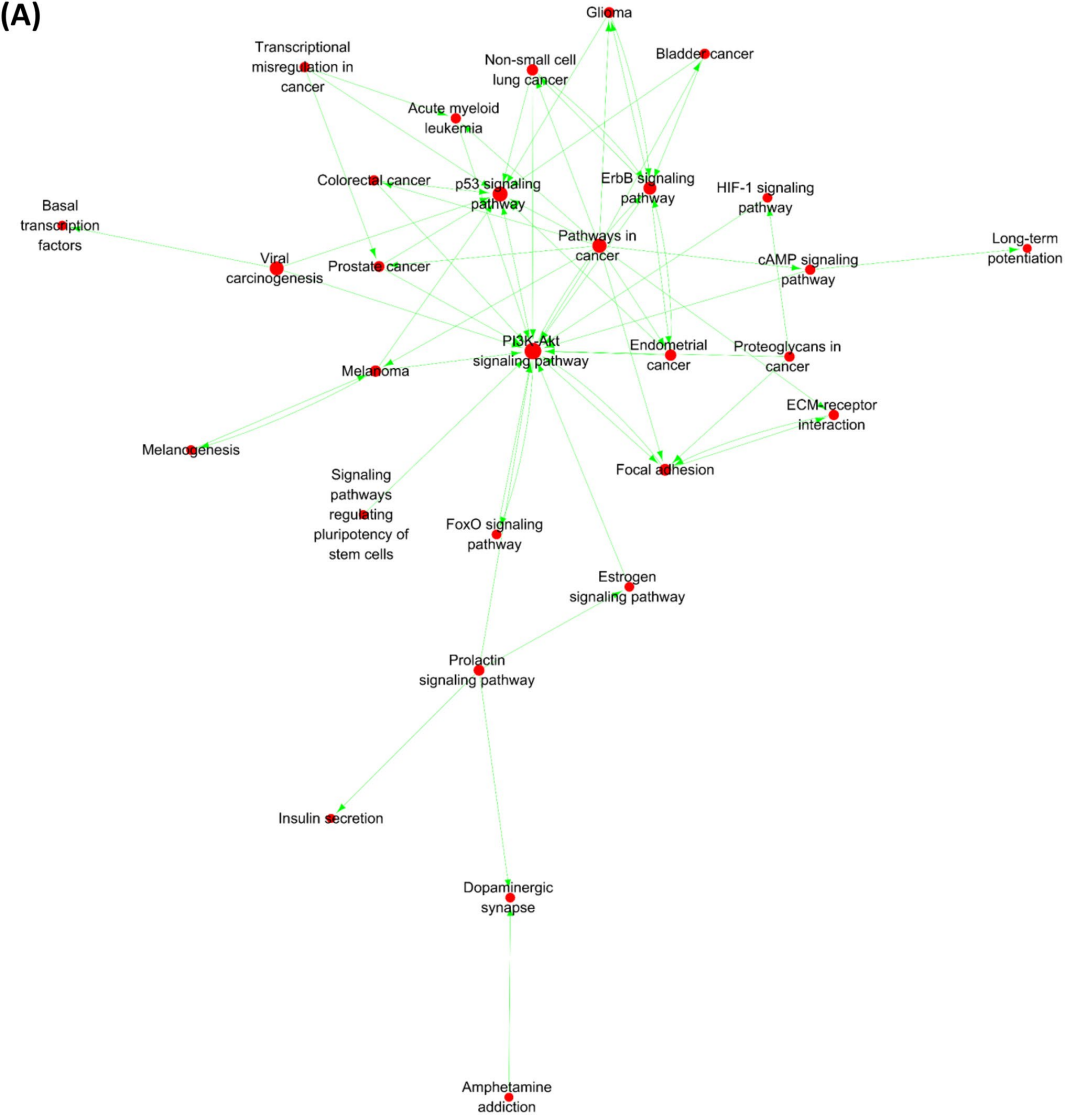

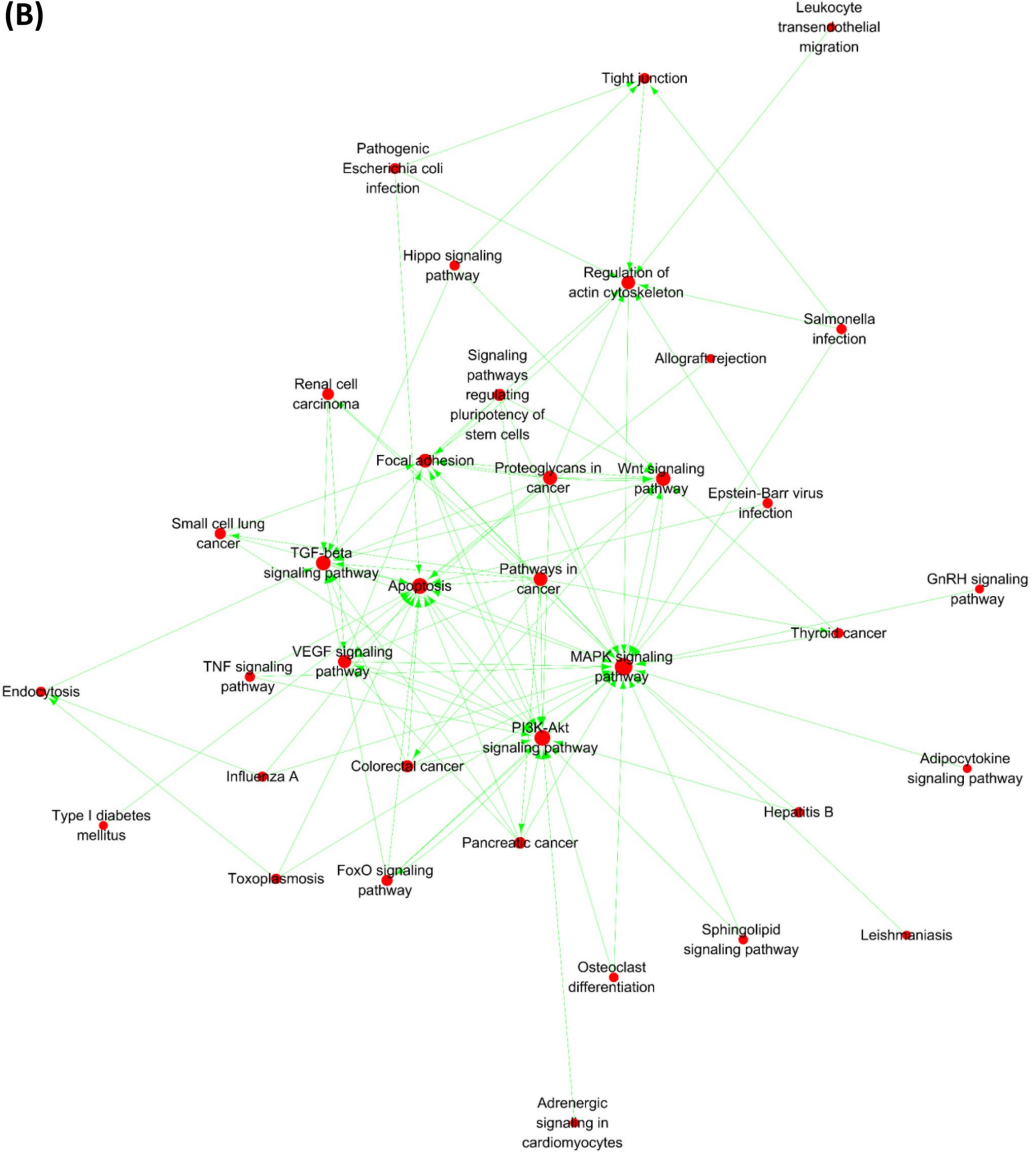
(**A**–**B**) The interaction network of significant pathway (Path-Net) is a functional mapping analysis according to KEGG pathway analysis to visualize the differentially upregulated (**A**) and downregulated (**B**) expression of SE-associated mRNAs in different clusters. Nods represent different signaling pathways. Lines indicate a trigger relationship between significant pathways

In order to obtain the expression profile of SE-lncRNAs and their associated mRNAs, which overlapped or were closest to 50 kb of the transcription start site of SE-lncRNAs, we subjected total RNA isolated from leiomyoma and paired myometrium (*N* = 8) to microarray analysis using the Arraystar SE-lncRNAs Arrays. Thousands of SE-lncRNAs and coding transcripts were successfully detected by microarray probes in our paired specimens. Following normalization, the analysis comparing leiomyomas with the matched myometrium group resulted in identification of 7680 SE-lncRNAs, of which 721 SE-lncRNAs were upregulated and 247 SE-lncRNAs were downregulated by 1.5-fold or greater in leiomyoma. For associated mRNAs within 50 kb of the SEs, 6917 were identified, of which 539 mRNAs were upregulated, while 712 mRNAs were downregulated in leiomyoma group by 1.5-fold or greater. Hierarchical clustering and TreeView analysis of SE-lncRNAs (Fig. 1A) and SE-associated mRNAs (Fig. 1B) revealed clusters which were separated into their respective groups. As shown in Fig. 2, volcano plot filtering indicated that 581 SE-lncRNAs were upregulated and 179 SE-lncRNAs were downregulated in leiomyomas compared with matched myometrium (fold change > 1.5 and *P* < 0.05) (Fig. 2A). In contrast, 427 mRNAs were upregulated, while 588 mRNAs were downregulated (Fig. 2B). Scatter plots were used to visualize the variable relationships between leiomyomas and matched myometrium which showed a 1.5-fold change in abundance of SE-lncRNAs (Fig. 2C) and mRNAs (Fig. 2D). The GO analysis indicated ten up- and downregulated mRNAs with top ten enrichment scores covering categories of biological processes, cellular components, and molecular functions (Fig. 3A and B). The KEGG enrichment analysis disclosed ten pathways correlated with the up- and downregulated mRNAs. Among the upregulated coding transcripts the signaling pathway responsible for proteoglycans (PG) regulation was the most significantly enriched (Fig. 3C). Other pathways pertinent to leiomyoma pathogenesis were the estrogen signaling and PI3K-AKT signaling pathway. KEGG enrichment analysis of the downregulated coding transcripts revealed Th1, Th2, and Th17 (type 1, type 2, and type 17 helper T cells) cell differentiation was the most significantly altered pathways in leiomyomas (Fig. 3D). Based on the enrichment pathway analysis, the map of the corresponding pathway-gene networks was constructed and shown in Fig. 4A and B.

### Validation of SE-lncRNAs and Their Corresponding Protein Coding Gene Expression in Leiomyoma and Matched Myometrium

**Fig. 5:**
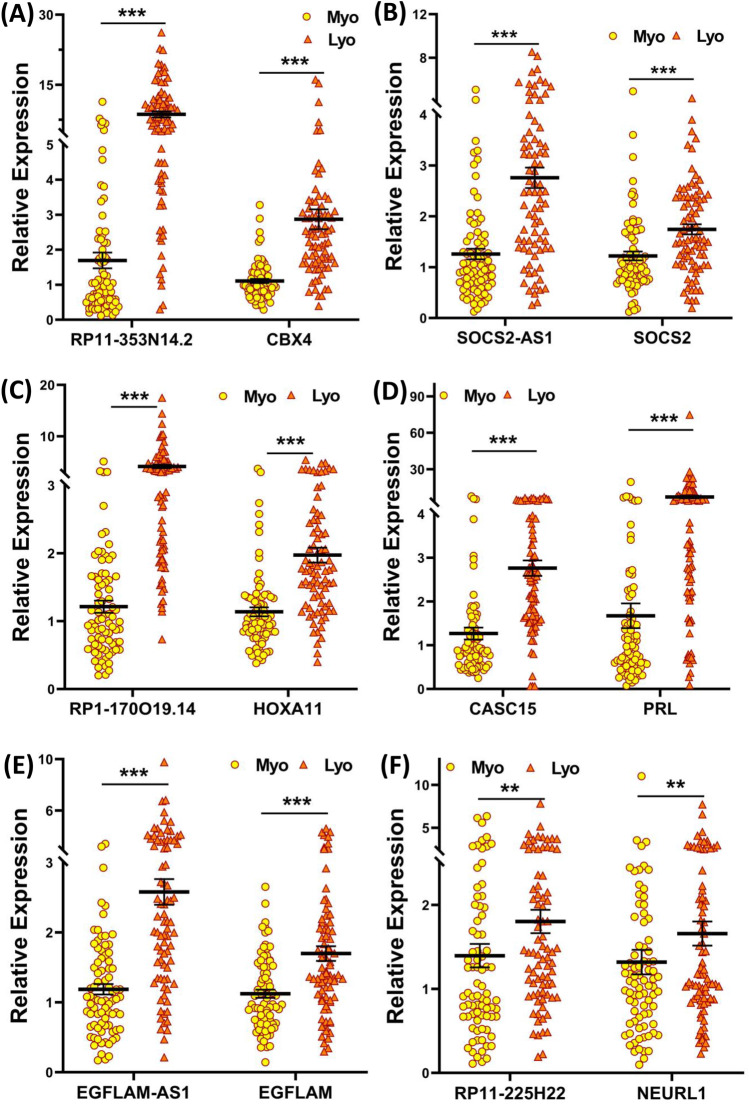

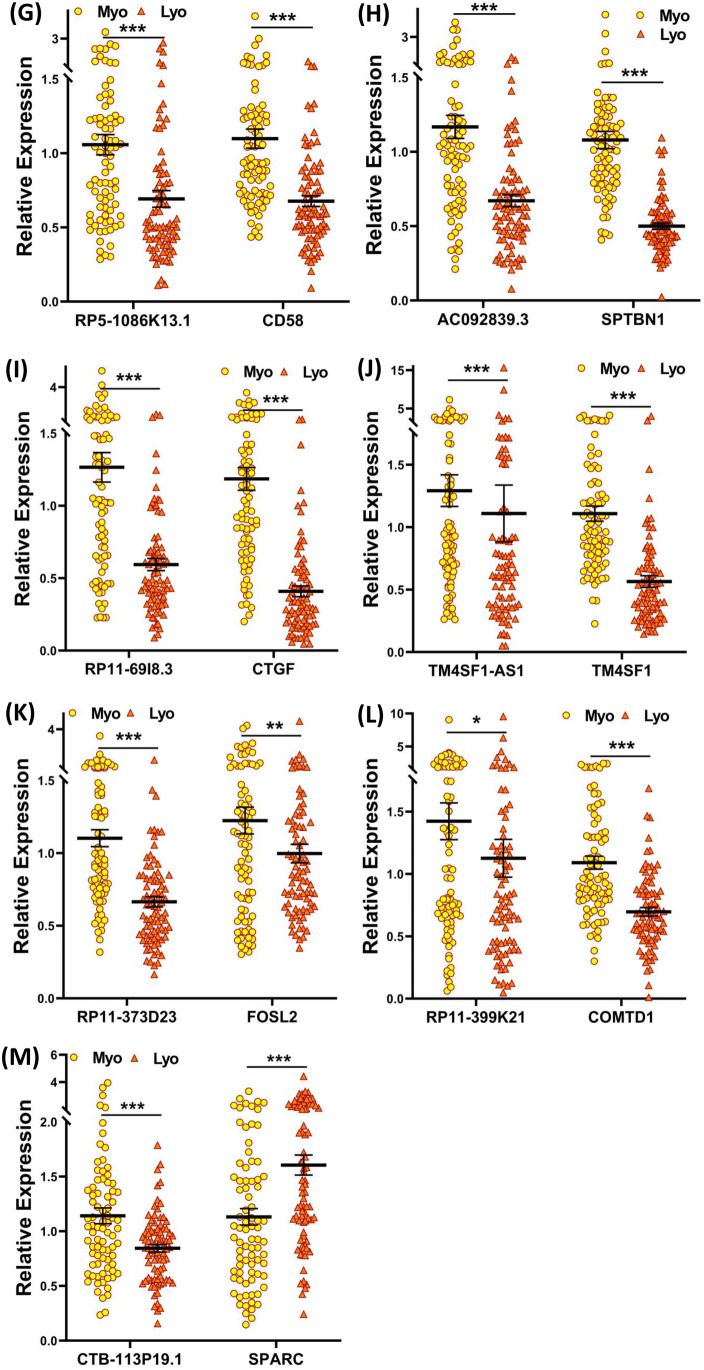
The expression of selected SE-lncRNAs **A** RP11-353N14.2/CBX4, **B** SOCS2-AS1/SOCS2, **C** RP1-170O19.14/HOXA11, **D** CASC15/PRL, **E** EGFLAM-AS1/EGFLAM, **F** RP11-225H22/NEURL1, **G** RP5-1086K13.1/CD58, **H** AC092839.3/SPTBN1, **I** RP11-69I8.3/CTGF, **J** TM4SF1-AS1/TM4SF1, **K** RP11-373D23/FOSL2, **L** RP11-399K21.11/COMTD1, and **M** CTB-113P19.1/SPARC in eighty-one paired leiomyoma tissues by qRT-PCR. The results are presented as mean ± SEM with *P* values (*P < 0.05; **P < 0.01; ***P < 0.001) indicated by corresponding lines

In order to validate the SE-lncRNAs array data, we selected thirteen pairs of SE-lncRNAs and their corresponding protein coding transcripts for further analysis by qRT-PCR in eighty-one paired leiomyoma specimens (Table 1 and Fig. 5). The selection of these SE-lncRNAs and their corresponding protein coding transcripts was based on their differential expression by microarray analysis in leiomyoma as well as prior publications demonstrating their variable expression in a number of disorders and their involvement in epigenetic regulation, extracellular matrix deposition, and tumorigenesis all of which are hallmarks of leiomyoma pathogenesis and progression [1–4]. Among the thirteen SE-lncRNAs analyzed, six SE-lncRNA and their corresponding coding transcripts were both significantly upregulated in leiomyomas (RP11-353N14.2/CBX4, SOCS2-AS1/SOCS2, RP1-170O19.14/HOXA11, CASC15/PRL, EGFLAM-AS1/EGFLAM, RP11-225H22/NEURL1) (Fig. 5A–F), while six SE-lncRNAs with their corresponding protein coding genes were both significantly downregulated in leiomyomas (RP5-1086K13.1/CD58, AC092839.3/SPTBN1, RP11-69I8.3/CTGF, TM4SF1-AS1/TM4SF1, RP11-373D23/FOSL2, and RP11-399K21.11/COMTD1) (Fig. 5G–L). However, the expression of CTB-113P19.1 was decreased in leiomyomas, while its overlapping protein coding gene, SPARC, was upregulated (Fig. 5M). Overall, the qRT-PCR results validated the microarray analysis.

**Table 1:** The background information of the thirteen SE-lncRNAs with their associated transcripts validated in this study

SE-lncRNAs		Overlapped	Proximal	
Gene symbol	Location	Gene symbol	Gene symbol	Location
RP11-353N14.2	chr17:79823452–79827704 (GRCh38/hg38)		CBX4	chr17:79,833,156–79,839,440 (GRCh38/hg38)
SOCS2-AS1	chr12:93,503,696–93,571,768 (GRCh38/hg38)	SOCS2		chr12:93,569,814–93,626,236 (GRCh38/hg38)
RP1-170O19.14	chr7:27186573–27193448 (GRCh38/hg38)		HOXA11	chr7:27,181,157–27,185,232 (GRCh38/hg38)
CASC15	chr6:21,665,003–22,214,734 (GRCh38/hg38)		PRL	chr6:22,287,244–22,302,897 (GRCh38/hg38)
EGFLAM-AS1	chr5:38,425,036–38,427,376 (GRCh38/hg38)	EGFLAM		chr5:38,258,409–38,465,480 (GRCh38/hg38)
RP11-225H22	chr10:103,479,602–103,517,442 (GRCh38/hg38)	NEURL1		chr10:103,493,705–103,592,552 (GRCh38/hg38)
RP5-1086K13.1	chr1:116493023–116499212 (GRCh38/hg38)		CD58	chr1:116,500,390–116,571,039 (GRCh38/hg38)
AC092839.3	chr2:54516048–54540697 (GRCh38/hg38)	SPTBN1		chr2:54,456,317–54,671,446 (GRCh38/hg38)
RP11-69I8.3	chr6:131,950,946–132,077,393 (GRCh38/hg38)	CTGF		chr6:131,948,176–131,951,372 (GRCh38/hg38)
TM4SF1-AS1	chr3:149,377,778–149,386,583 (GRCh38/hg38)	TM4SF1		chr3:149,369,022–149,377,692 (GRCh38/hg38)
RP11-373D23	chr2:28,425,945–28,426,719 (GRCh38/hg38)		FOSL2	chr2:28,392,448–28,417,317 (GRCh38/hg38)
RP11-399K21	chr10:75,397,830–75,401,764 (GRCh38/hg38)		COMTD1	chr10:75,233,641–75,236,030 (GRCh38/hg38)
CTB-113P19.1	chr5:151,676,944–151,726,300 (GRCh38/hg38)	SPARC		chr5:151,661,096–151,686,975 (GRCh38/hg38)

**Fig. 6:**
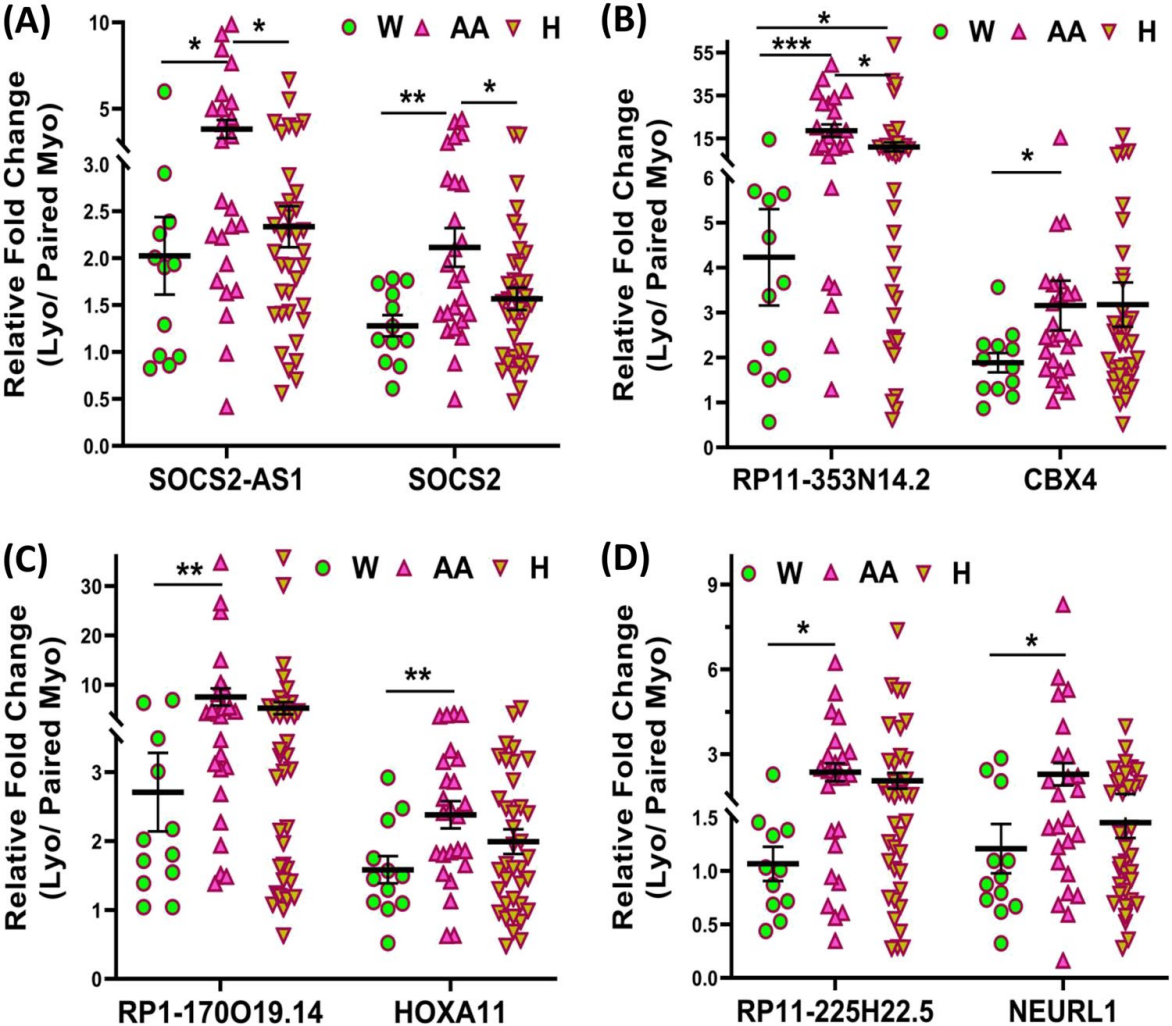
The expression of **A** SOCS2-AS1/SOCS2, **B** RP11-353N14.2/CBX4, **C** RP1-170O19.14/HOXA11, and **D** RP11-225H22/NEURL1 expressed as fold change (Lyo/paired Myo) based on race/ethnicity in Caucasian (W; n=12), African American (AA; n=25), and Hispanic (H; n=37) by qRT-PCR. The results are presented as mean ± SEM with *P* values (*P < 0.05; **P < 0.01; ***P < 0.001) indicated by corresponding lines

**Fig. 7:**
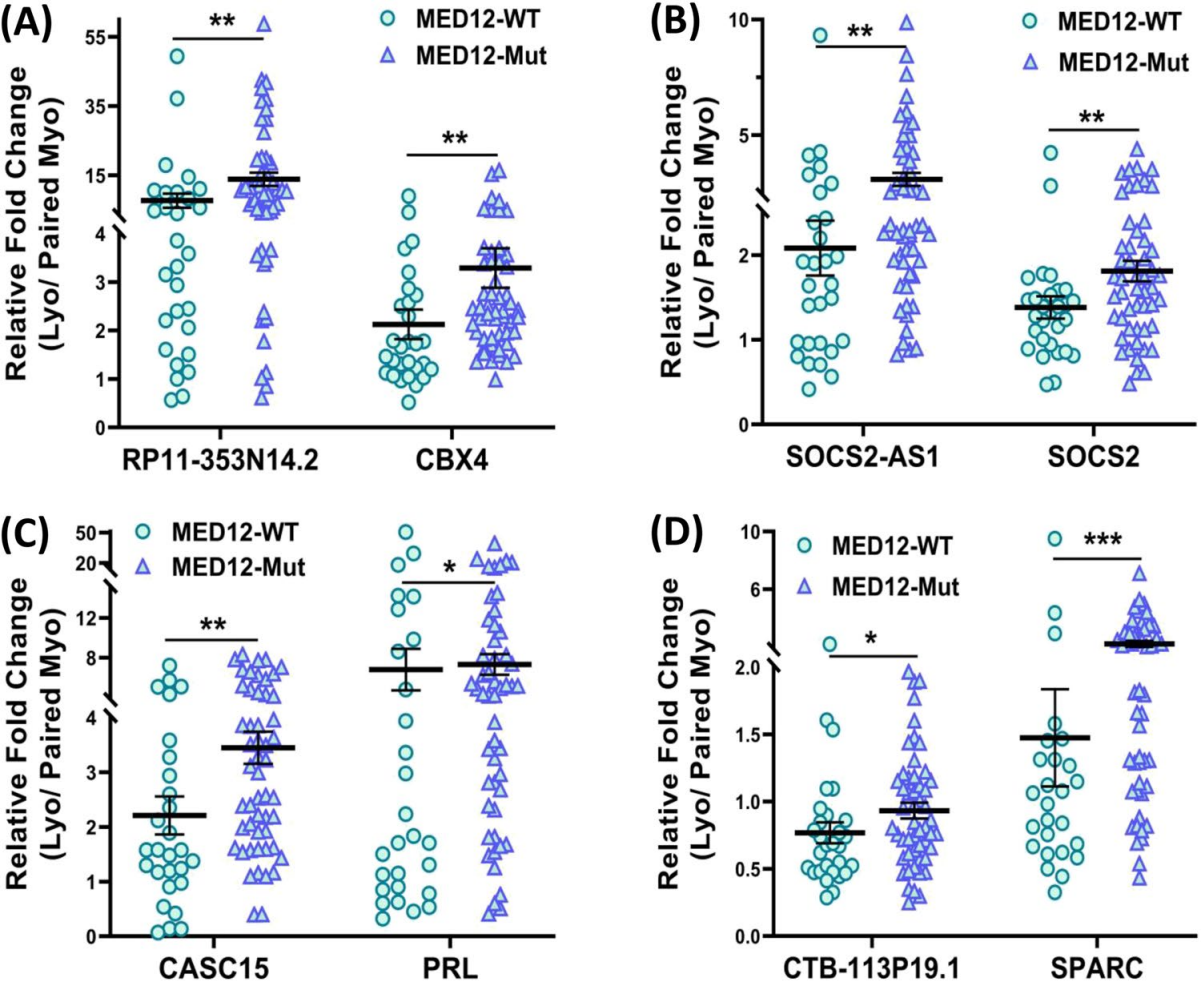
The expression of **A** RP11-353N14.2/CBX4, **B** SOCS2-AS1/SOCS2, **C** CASC15/PRL, and **D** CTB-113P19.1/SPARC expressed as fold change (Lyo/paired Myo) based on MED12 mutation status in mutation negative tumors (MED12-WT; n=29) and MED12 bearing mutations (MED12-Mut; n=52) by qRT-PCR. The results are presented as mean ± SEM with *P* values (*P < 0.05; **P < 0.01; ***P < 0.001) indicated by corresponding lines

Next, we analyzed the expression of SE-lncRNAs and their associated mRNAs which were most significantly dysregulated based on fold change (leiomyoma vs. matched myometrium) in different racial groups and in tumors with and without the MED12 mutation. This analysis indicated that the expression of SOCS2-AS1/SOCS2, RP11-353N14.2/CBX4, RP1-170O19.14/HOXA11, and RP11-225H22/NEURL1 was significantly higher in African Americans as compared with Caucasians (Fig. 6A–D). In the case of leiomyomas from the Hispanic patients, the only significant difference was in the expression of the pair SOCS2-AS1/SOCS2 which was significantly higher in African Americans as compared with the Hispanics (Fig. 6A). The mutation analysis of the specimens indicated that 52 of the 81 specimens had the MED12 mutation. Missense mutations in MED12 exon 2 were the most frequent alteration *(*42/52 pairs*),* followed by in-frame insertion–deletion-type mutations (10/52 pairs*).* The missense mutations in exon 2 included c.130G > C (p.Gly44Arg) (5/42 pairs), c.130G > A (p.Gly44Ser) (8/42 pairs), c.130G > T (p.Gly44Cys) (3/42 pairs), c.131G > C (p.Gly44Ala) (2/42 pairs), c.131G > A (p.Gly44Asp) (17/42 pairs), c.131G > T (p.Gly44Val) (6/42 pairs), and c.128A > C (p.Gln43Pro) (1/42 pairs). In terms of MED12 mutation status analysis, the expression of four SE-lncRNAs and their corresponding coding transcripts (RP11-353N14.2/CBX4, SOCS2-AS1/SOCS2, CASC15/PRL, CTB-113P19.1/SPARC) was significantly higher in tumors bearing the MED12 mutation as compared with MED12-mutation-negative tumors (Fig. 7A–D).

## Discussion

This study for the first time evaluated the expression profile of SE-lncRNAs and associated mRNAs within 50 kb of the SE-lncRNAs transcription start site in leiomyomas. The microarray analysis showed dysregulation of a host of SE-lncRNAs and their associated mRNAs in leiomyomas. We found that aberrant expression of SE-lncRNAs was distributed across all the chromosomes including the X chromosome. In additional analysis, we selected thirteen SE-lncRNAs and their associated mRNAs and determined their expression by qRT-PCR in eighty-one paired leiomyomas and myometrium. The qRT-PCR analysis validated the microarray results, demonstrating differential expression of these SE-lncRNAs in leiomyomas. KEGG pathway analysis of the differentially expressed SE-associated mRNAs revealed that multiple signaling pathways such as proteoglycan synthesis pathway, estrogen signaling, PI3K-AKT, TGF-β (transforming growth factor beta) signaling, and the signaling pathways involving Th1, Th2, and Th17 cell differentiation which are all relevant to leiomyoma pathogenesis were altered in leiomyomas. These novel findings indicate that dysregulation of SE-lncRNAs in leiomyomas could influence the expression of a host of nearby coding genes previously shown by several groups to be key to leiomyoma pathogenesis.

The traditional view of gene regulation has focused on protein-coding genes; however, this view has changed since the discovery of non-coding RNAs such as miRNAs and lncRNAs [23, 24, 40, 41]. The abnormal expression of protein coding genes critical in leiomyoma pathogenesis is regulated by numerous molecules and signaling pathways [1–4], which in turn are under the regulation of miRNAs and lncRNAs [16–20, 30]. Recent studies have reported that SEs are large clusters of enhancers in close genomic proximity that are involved in chromatin three-dimensional structures and epigenetic histone modifications [42–44]. SE-lncRNAs, a class of lncRNAs transcribed from SE regions, can play essential roles in transcriptional regulation of SE activity to drive the expression of genes which regulate cellular development and differentiation. The expression of SE-lncRNAs is dysregulated in multiple diseases such as cancer and lung fibrosis [45–49].

Our data indicates that a number of dysregulated protein coding genes in leiomyomas previously shown by various groups [1–4] could be under the transcriptional regulation of SE-lncRNAs. One such pathway identified by KEGG analysis as the most affected is the PG synthesis pathway. PG is a component of the ECM which is highly accumulated in leiomyomas [50]. PGs are cross-linked to interstitial collagens within the ECM and contribute to leiomyoma stiffness [50]. Other pathways that were upregulated are the estrogen signaling which is well established as a promoter of growth of these tumors [1–4] and the PI3K-AKT signaling previously reported to be dysregulated in leiomyomas [1–4]. The potential significance of the downregulated Th1, Th2, and Th17 cell differentiation pathways and the PD-L1 (programmed death-ligand 1)/PD-1 (programmed cell death protein 1) checkpoint pathway may be related to possible immune evasion by these tumors similar to malignant ones [51]. The downregulation of TGF-β pathway was unexpected as several groups including ours have reported upregulation of this pathway in leiomyomas and a significant mechanism underlying the fibrosis associated with these tumors [1, 2, 37]. Further functional studies are needed to determine the role of these SE-lncRNAs in regulating genes known to be dysregulated in leiomyomas.

One of the differentially expressed SE-lncRNAs identified in this study and its associated protein-coding genes, TM4SF1-AS1, has been investigated in several malignant tumors. In hepatocellular carcinoma (HCC), TM4SF1-AS1 and its associated protein were overexpressed and found to be responsible for hypoxia-enhanced cell proliferation, mobility, and invasive abilities of HCC cells [52]. TM4SF1-AS1 expression was also induced in gastric cancer tissues and was shown to increase cell proliferation, invasion, and the epithelial-to-mesenchymal transition through regulation of TM4SF1 levels and PI3K-AKT signaling pathways [53]. TM4SF1 is a highly expressed cell surface antigen which mediates signal transduction events that facilitate cell development, growth, invasion, and metastasis in different carcinomas [54, 55]. This study identified significant downregulation of TM4SF1-AS1 and TM4SF1 in leiomyomas which might be a potential mechanism underlying the non-invasiveness of these tumors. Another differentially expressed protein coding transcript was CBX4. CBX4 is a component of PRC1 (polycomb repressive complex 1), known as an epigenetic regulator complex regulating cell differentiation, proliferation, and senescence through PRC1-dependent and PRC1-independent mechanisms [56]. CBX4 has been reported to be involved in uterine stromal cell decidualization through transcriptional regulation of genes related to extracellular remodeling [57]. CBX4 also acts as a SUMO-E3 ligase for modulation of the activity and stability of DNMT3A [58], which is overexpressed in leiomyomas [18]. Some of the altered genes were found to be associated with cancer progression and prognosis. For example, higher levels of EGFLAM and FOSL2 have been reported to correlate with poor prognosis and therefore considered as prognostic biomarkers and therapeutic targets in some cancers [59–63]. Higher expression of CTB-113P19.1 is correlated with liver and lymph node metastasis in colorectal cancer patients with overall shorter survival duration [64]. Although the expression of CTB-113P19.1 was lower in leiomyomas, its overlapped transcript SPARC was significantly higher. The cis-acting lncRNAs can activate, repress, or modulate the expression of target genes through various mechanisms, such as recruitment of transcriptional repressors, acting as antisense against nearby transcripts, and competition with nearby available enhancers and thereby interference with transcription [65].

SPARC is a phosphorylated, cysteine-rich acidic, glycine-rich glycoprotein that is present in large amounts in human placenta and is involved in extracellular matrix synthesis and is required for the collagen in bone calcification [66]. SPARC has been associated with tumor metastasis based on the alteration of cell shape which facilitates tumor invasion [66]. Because SPARC regulates cell–matrix interactions and also regulates the activity of many growth factors, such as fibroblast growth factor (FGF)-2, vascular endothelial growth factor (VEGF), and platelet-derived growth factor (PDGF) [4], it could play a significant role in leiomyoma pathogenesis. SPTBN1 is a TGF-β signal-transducing adapter protein which is necessary for Smad3/Smad4 complex formation and functions as a tumor suppressor in many cancers [67, 68]. Our data showed downregulation of SPTBN1 in leiomyomas; reduced expression of SPTBN1 correlated with shorter survival of patients with cancer [67, 68]. The expression of EGFLAM-AS1 in leiomyomas was upregulated; EGFLAM-AS1 is a tumor suppressor in many cancers through its targeting leukemia inhibitory factor receptor (LIFR) which is a known metastasis inhibitor [69]. NEURL1 is a highly conserved E3 ubiquitin ligase which was reported to be a tumor suppressor in medulloblastoma through its regulation of Jagged1 levels to influence the Notch signaling pathway [70, 71]. Our study indicated increased expression levels of RP11-225H22 and its overlapped transcript NEURL1 in leiomyomas which may contribute to the reduced levels of Jagged1 reported in leiomyomas [72]. We also found a decrease in the expression of RP5-1086K13.1 and its proximal transcript CD58 in leiomyomas which in colorectal cancer cells were found to induce the Wnt/β-catenin pathway [73]; this pathway is known to be activated in leiomyomas [1]. CTGF is a cysteine-rich growth factor involved in many biological processes such as cell proliferation, adhesion, migration, and matrix production and plays a critical role in several fibrotic diseases and cancers [74, 75]. Our data indicated a significant decrease in SE-lncRNA RP11-69I8.3 expression and its overlapping gene, CTGF in leiomyomas. Previous studies also reported the downregulation of CTGF in leiomyomas [4, 76]. COMTD1, a putative O-methyltransferase, has been shown to be differentially methylated in schizophrenia patients, leading to its altered transcription [77]. Although our study showed decreased expression of COMTD1 mRNA in leiomyomas, another member of the methyltransferase superfamily, COMT, (catechol-O-methyltransferase) which is involved in metabolism of steroid hormones was reported to be overexpressed in uterine leiomyomas with higher frequency of COMT Val158Met genotype in African Americans [78, 79]. The dysregulation of this gene was proposed as a potential mechanism underlying racial differences in leiomyoma severity [78, 79].

SOCS2 is a cytokine-inducible negative regulator of cytokine signaling which was reported to be overexpressed in leiomyomas as compared to scar tissues [5]. Our data also demonstrated an upregulation of SOCS2 expression in leiomyomas. The functional role of SOCS2 in leiomyomas has yet to be determined. SOCS2 knockout mice exhibited growth acceleration resulting in 1.3 ~ 1.5 times the size of adult mice [80]. The expression of SOCS2 was induced by a subset of cytokines such as interferon-γ, GM-CSF, and IL-10, and it influenced IGF1R (insulin-like growth factor 1 receptor)-mediated cell signaling [81]. The overlapped SE-lncRNA SOCS2-AS1 correlated positively with SOCS2 expression and was a poor prognosis marker when its expression was lower in colorectal cancer patients [82]. Conversely, the expression of SOCS2-AS1 was increased in castration-resistant prostate cancer model cells where it induced androgen signaling through an epigenetic mechanism involving the regulation of AR target genes and resulted in castration-resistant and androgen-dependent cell growth [83]. The SE-lncRNA RP1-170O19.14 was upregulated in gastric cancer [84]. Its proximal associated gene HOXA11 was decreased in endometrium of patients with submucosal and intramural leiomyomas [85, 86]. Our data indicated increased expression of RP1-170O19.14 and its proximal gene HOXA11 in leiomyomas. Finally, CASC15 expression was upregulated in many cancers and a potential hotspot for chromosome alterations in MED12-mutation-negative leiomyomas [7, 87]. PRL, the associated protein-coding gene of CASC15, is one of the most highly expressed genes in leiomyomas [88]. PRL activated MAPK and STAT5 signaling in human myometrial cell lines and promoted myofibroblast trans-differentiation which in turn contributed to leiomyomas fibrosis [89]. Our data also indicated an upregulation of PRL in leiomyomas which is in line with prior reports and a potential mechanism for MAPK activation in leiomyomas [89, 90]. Overall, the expression of the selected SE-lncRNAs in this study correlated with their corresponding protein coding transcripts many of which are known to be pivotal in leiomyoma pathogenesis.

Race plays an important role in leiomyoma severity and progression with African Americans having greater severity of symptoms and earlier onset [1–4]. Intense efforts have been focused to determine the mechanism underlying this racial disparity. Here, we showed that the expression levels of four SE-lncRNAs and their associated transcripts significantly correlated with race/ethnicity (RP11-353N14.2/CBX4, SOCS2-AS1/SOCS2, RP1-170O19.14/HOXA11, and RP11-225H22/NEURL1). Because super-enhancers regulate the expression of a host of genes, the racial differences in a select group of super-enhancers as shown here could be an important mechanism underlying racial differences in leiomyoma development and progression. Previous studies have provided evidence for potential mechanisms underlying the racial disparity in tumor development and progression including polymorphism of the genes COMT, ER (estrogen receptor), and CYP17 (cytochrome P450C17α) which are involved in estrogen synthesis, function and/or metabolism, and the upregulation of aromatase (CYP19) gene in tumors of African American women as compared with Caucasians [6, 79]. Tumors derived from African Americans were also reported to express higher levels of progesterone receptor A (PR-A) and lower levels of retinoic acid receptor-α (RARA) [6, 79]. Furthermore, African American women have higher incidence of vitamin D deficiency which is a risk factor for leiomyoma development [6, 79]. A previous report using microarray analysis showed significant alteration in the expression of transcription factors (ZFX (zinc finger X-chromosomal protein), NFIB (nuclear factor 1 B-type)) and genes involved in transcription (GABPA (GA-binding protein alpha chain), STAT2 (signal transducer and activator of transcription 2), MXD4 (max-interacting transcriptional repressor), IKBKG (inhibitor of nuclear factor kappa-B kinase subunit gamma), NFATc4 (nuclear factor of activated T cells, cytoplasmic 4)), translation (EIF5A (Eukaryotic translation initiation factor 5A-1), EIF5 (eukaryotic translation initiation factor 5)), and RNA processing (DDX6 (probable ATP-dependent RNA helicase)) in African Americans [91]. Recent reports from our laboratory revealed dysregulation of tryptophan catabolism in leiomyomas, with tumors of African American women expressing more TDO2 (tryptophan 2,3-dioxygenase) mRNA but lower levels of KYNU (L-Kynurenine hydrolase) mRNA as compared to Caucasian women [38, 92]. Moreover, the expression of several miRNAs, such as miR-200c, miR-21, miR-23b, and Let-7 s, was dysregulated significantly more in leiomyomas of African American as compared with Caucasian women [36, 93].

The most common driver mutations in leiomyomas are those involving MED12. We found that the expression of a number of SE-lncRNAs (RP11-353N14.2/CBX4, SOCS2-AS1/SOCS2, CASC15/PRL, CTB-113P19.1/SPARC) was MED12 mutation-dependent. This finding suggests that the mediator complex regulates the transcription of some of the SE-lncRNAs identified here. Current evidence indicates that SEs are large clusters of enhancers harboring unusually high level of transcription factors, epigenetic modifications (e.g., H3K27ac and H3K4me1/2) and recruit co-regulators (e.g., acetyltransferase CBP/p300, and mediator complex) [42–44]. MED12 is a component of mediator complex which functions as a transcription coactivator by transmitting signals from gene-specific transcription factors to RNA polymerase II, resulting in either promotion or repression of gene transcription [94]. The knockdown of MED12 only affected the expression of genes adjacent to SEs as compared to those adjacent to typical enhancers [44, 95].

In summary, we identified a number of differentially expressed SE-lncRNAs and their associated mRNAs in leiomyomas. Furthermore, we selected and verified the co-expression of SE-lncRNAs and their corresponding protein coding transcripts. Our findings implicate SE-lncRNAs in leiomyoma tumorigenesis and progression through the regulation of potential downstream target genes which are in close proximity to their transcription sites (e.g., SOCS2, CBX4, HOXA11, PRL, SPARC, and EGFLAM). The expression of some of these SE-lncRNAs correlate with race/ethnicity and MED12 mutation status of the tumor. Functional studies are underway in our laboratory to elucidate the physiological significance of these SE in leiomyomas pathogenesis.

## Supplementary Information

Below is the link to the electronic supplementary material.

Supplementary file1 (XLSX 11 KB)

## Data Availability

Raw data were generated at the Lundquist Institute. Derived data supporting the findings of this study are available from the corresponding author O. K. on request.
